# Myobiid mites (Trombidiformes, Myobiidae) of the golden bat *Mimon cozumelae* from Mexico. Description of the male and tritonymph of *Ioanella mimon* and new records of *Eudusbabekia mimon*

**DOI:** 10.3897/zookeys.658.11507

**Published:** 2017-02-22

**Authors:** Angel Herrera-Mares, Carmen Guzmán-Cornejo, Livia León-Paniagua, Gerardo Rivas

**Affiliations:** 1 Laboratorio de Acarología, Departamento de Biología Comparada; 2 Museo de Zoología, Departamento de Biología Evolutiva, Facultad de Ciencias, Universidad Nacional Autónoma de México. Circuito Exterior s/n, Coyoacán, Ciudad Universitaria, 04510, Ciudad de México, México

**Keywords:** Myobiidae, *Ioanella*, *Eudusbabekia*, Phyllostomidae

## Abstract

The male and the tritonymph of *Ioanella mimon* are described for the first time parasitizing to *Mimon cozumelae* from Yucatan, Mexico. Male of *Ioanella mimon* is characterized by the presence of legs I with the tibia and tarsus fused forming a small complex devoided of apical claws, legs II–IV with two claws, setae *vi* at level of anterior end of genital plate, genital plate rounded with an anterior projection, all intercoxal setae short; while the tritonymph is characterized by the presence of legs I unequal; legs II–IV with 2-1-1 claws, and posterior region of dorsal idiosoma with 3 pairs of cylindrical and toothed setae. Additionally, we include new locality and host records for *Eudusbabekia mimon* which was also found on *Mimon cozumelae*. Both species were described originally in association with *Mimon bennettii* at Bartica, Guyana.

## Introduction

The genera *Eudusbabekia* Jameson, 1971 and *Ioanella* Dúsbabek & Lukoschus, 1973, include species associated with Phyllostomidae bats. The former is conformed by 32 species (Morales-Malacara et al. 2011) and the latter includes only five species (Bochkov 2009).

Particularly *Eudusbabekia mimon* Fain, 1973 and *Ioanella mimon* (Fain, 1973) were recorded parasitizing to *Mimon bennettii* Gray, 1938 from Bartica, Guyana (Fain 1973). Type material of both species is deposited in the Natural History Museum of London. The objective of this work is to provide the first morphological description of the male and tritonymph of *Ioanella mimon*, and new host and locality records for both species associated with *Mimon cozumelae* Goldman, 1914 from Yucatan, Mexico.

## Methods

**Figure 1.**
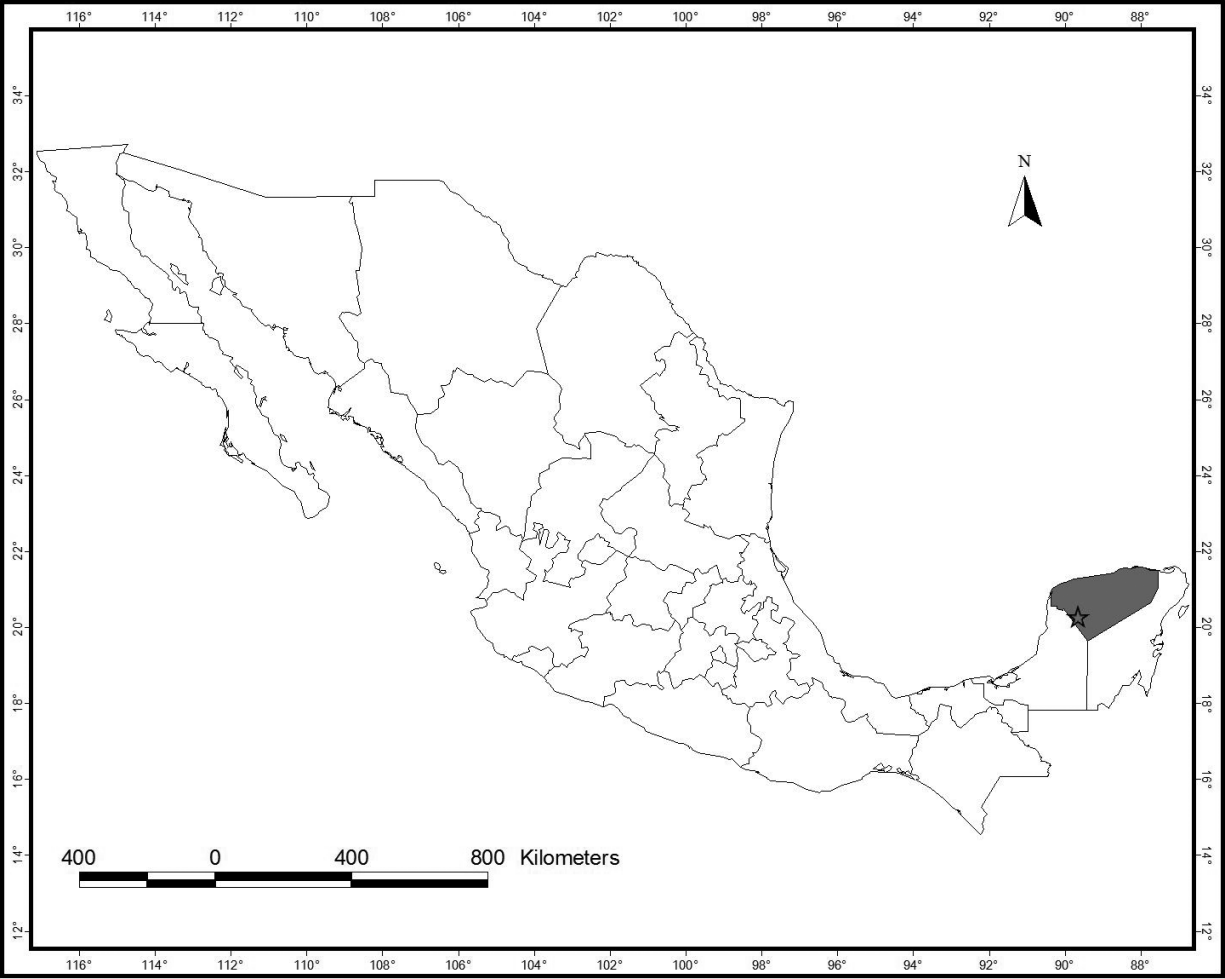
Map showing sampling site, carretera Santa Elena-Loltún, Km. 56, Yucatán, México.

A total of five bats were captured inside two hollowness located at carretera Santa Elena-Loltún Km 56, Yucatán, México (20°17'25.0"N, 89°38'43.3"W, 98 m) (Fig. 1). Bats were captured using mist nest and individually maintained until their posterior revision with a dissecting microscope. The Myobiidae (adults and nymphs) were removed from bats using fine, sharp forces and fixed and preserved in vials with 96% ethanol. The specimens were cleared in lactophenol and mounted in Hoyer’s medium. Mites were determined taxonomically. Descriptions and nomenclature for idiosomal setation follows Bochkov et al. (2008). Measures of body and setae are in micrometers and were made on a microscope Zeiss Axioscope 2 plus (Göttingen, Niedersachen, Germany), using the AXIOVISION 4 software; for measures we provide the average, followed by range in parenthesis. Drawings of specimens were made with a phase contrast microscope (Zeiss), equipped with a drawing tube. For the scanning electron microscopy (SEM), the specimens were dehydrated in 100% ethanol and dried to a critical point with liquid carbon dioxide. The dried specimens were mounted on aluminum specimen stubs, coated with a gold palladium alloy, and examined using a scanning electronic microscope Hitachi Stereoscan Model S–2469 N SEM (Hitachi Ltd., Tokyo, Japan). Mites were deposited at Colección del Laboratorio de Acarología, Facultad de Ciencias (LAFC), Universidad Nacional Autónoma de México (UNAM). Host were captured under the permission SGPA/DGVS/08257/13 and deposited at Colección de Mamíferos, Museo de Zoología “Alfonso L. Herrera”, Facultad de Ciencias (MZFC), UNAM.

## Taxonomy

### Family Myobiidae Mégnin, 1877

#### Eudusbabekia

##### Eudusbabekia mimon

Taxon classificationAnimaliaTrombidiformesMyobiidae

Fain, 1973

###### Material examined.

 1♂ ex *Mimon cozumelae*, Oquedad 1, carretera Santa Elena-Loltún Km. 56, Yucatán, México (LAFC-A01); 1♀, 1 PN same data, except Oquedad 2 (LAFC-A02).

#### *Ioanella* Dúsbabek & Lukoschus, 1973

##### Ioanella mimon

Taxon classificationAnimaliaTrombidiformesMyobiidae

(Fain, 1973)

Figs 2 , 3 , 4

###### Material examined.

7 TN, 3 ♀, ex *Mimon cozumelae*, Oquedad 1, carretera Santa Elena-Loltún Km 56, Yucatán, México (LAFC-A03); 4 TN, 3 ♀, 2♂, same data, except Oquedad 2 (LAFC-A04).

###### Description.

**Figure 2.**
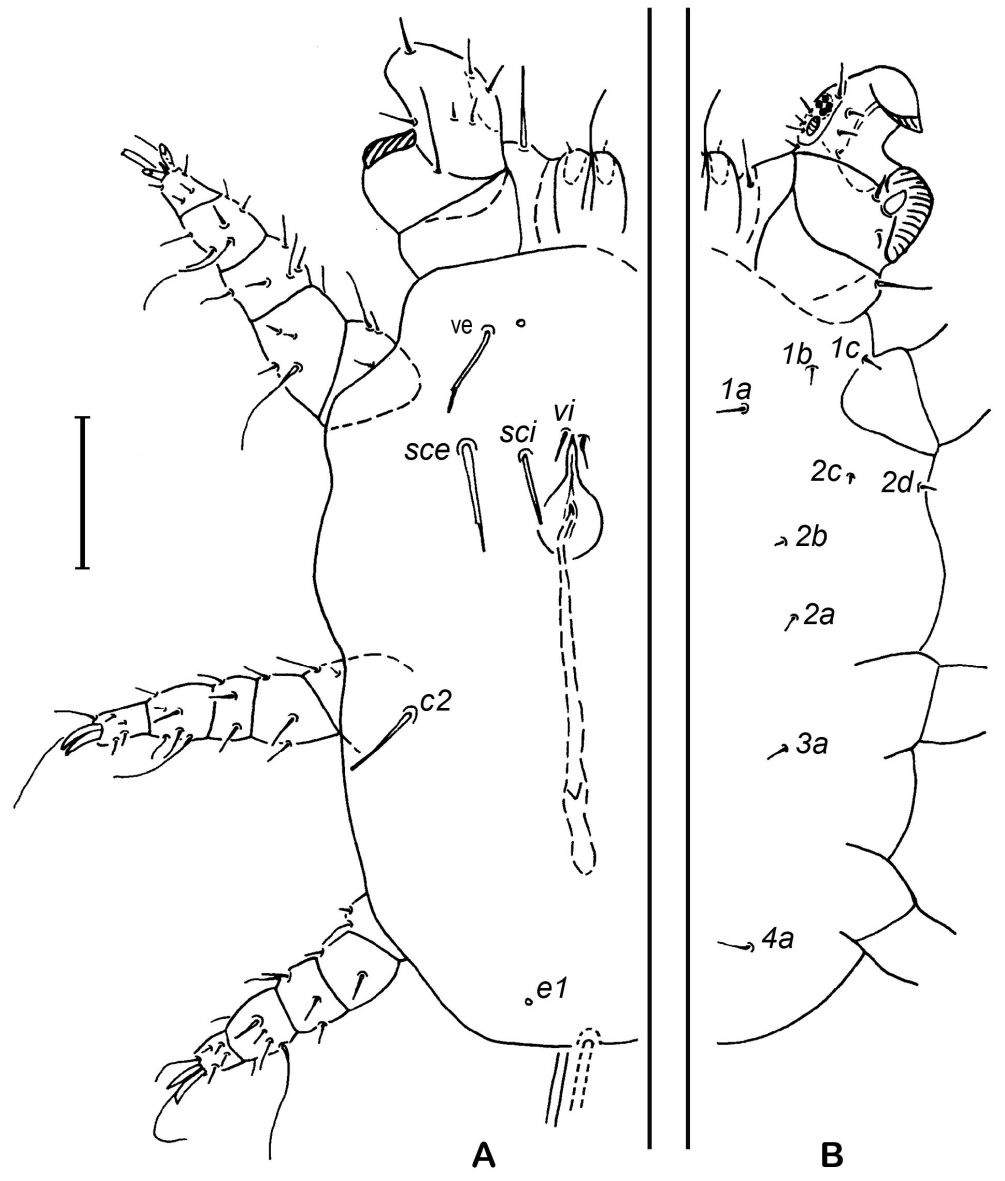
*Ioanella mimon*, male. **A** Dorsal view **B** Ventral view. Scale bar: 50 μm.

**Male (Based on 2 males).** Body length 225 (223–228); wide 139 (125–152). Body 1.6 larger than wide. Dorsal idiosoma (Fig. 2A). With a reduce number of setae. All dorsal setae slightly toothed except setae *vi*; *vi* at level of anterior end of genital plate; setae *sci* cylindrical, and situated close to the genital aperture. Setae *sce* cylindrical, with the base broad and becoming narrower to the tip and with the tip flat. Setae *c2* not distinctly inflated basally; *sci* situated at 15–16 behind the *sce*; setae *f2* absent as female; setae *e1* minute. Length of setae: *ve* 21 (18–25), *sce* 28 (26–31), *sci* 17 (14–17), *c2* 20 (17–22). Distances between bases of setae: *vi-vi*: 30 (29–31), *ve-ve*: 46 (45–47), *sce-sce*: 53 (52–54), *sci-sci*: 23 (21–26), *c2-c2*: 79 (76–82), *ve-sce* 28 (24–29), *sce-c2* 68 (65–68), *vi-sci* 31 (29–33). Genital plate rounded with an anterior projection (Fig. 2A). Penis 90 (90–91) long. Ventral idiosoma (Fig. 2B). All coxal setae filiform.

Gnathosoma. Normally developed, with a pair of ventral flat and retrorse processes as in the female (Fain, 1978) but slightly less pronounced.

**Figure 3.**
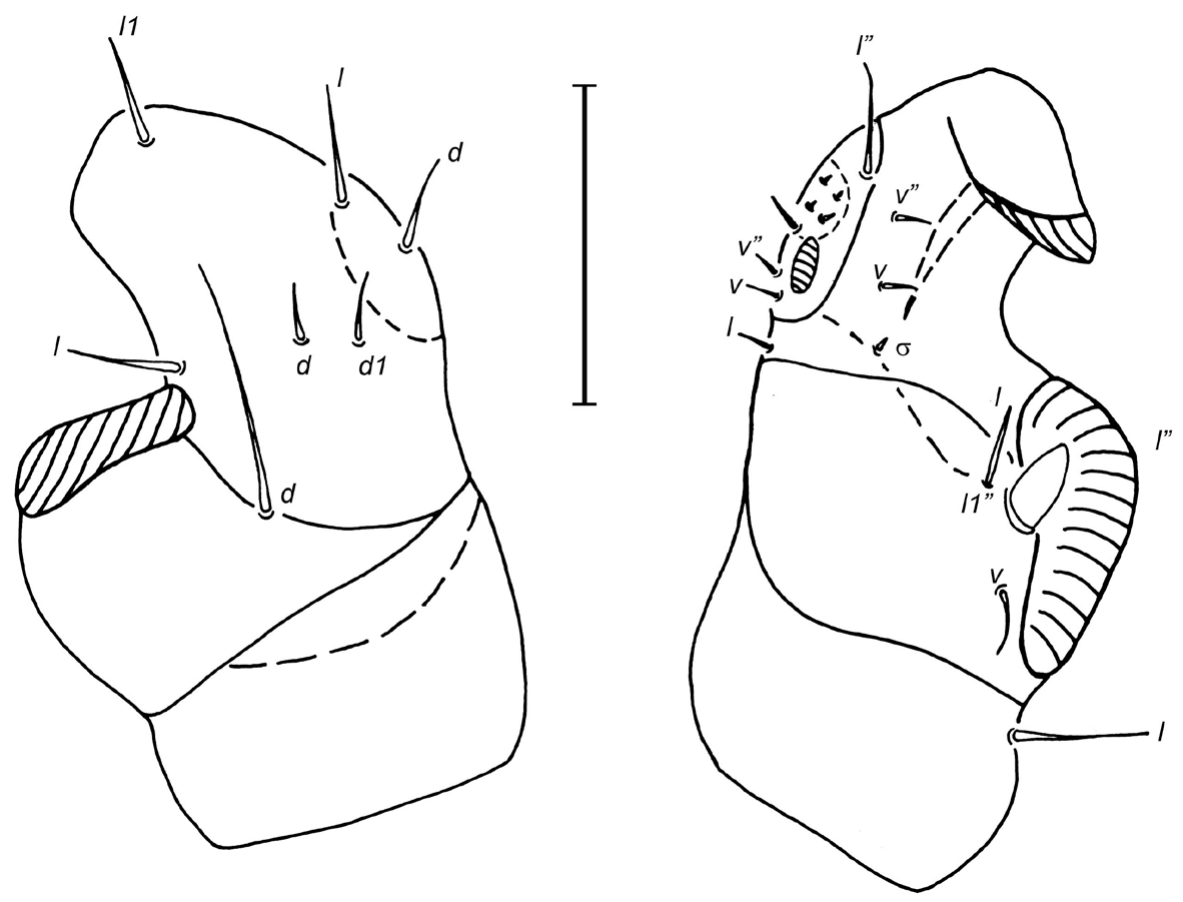
*Ioanella mimon*, male, leg I. **A** Dorsal view **B** Ventral view. Scale bar: 25 μm.

Legs. Tibia and tarsus I fused forming a small complex devoid of apical claws (Fig. 3). Genua I large, strongly oblique with a ventral clasping process recurved inwards and with 3 setae (Fig. 3). Trochanter I very broad, with the anterior end strongly expanded (Fig. 3). Legs II–IV narrow, ending in two short, subequal, and slightly curved claws. Setation for legs II–IV: tarsi 6-6-6, tibiae 6-6-6, genua 5-3-4, femora 5-3-2, trochanters 3-2-2. Tibia II–IV with a long and sinuous seta and a little thorn-like seta.

###### Description.

**Figure 4.**
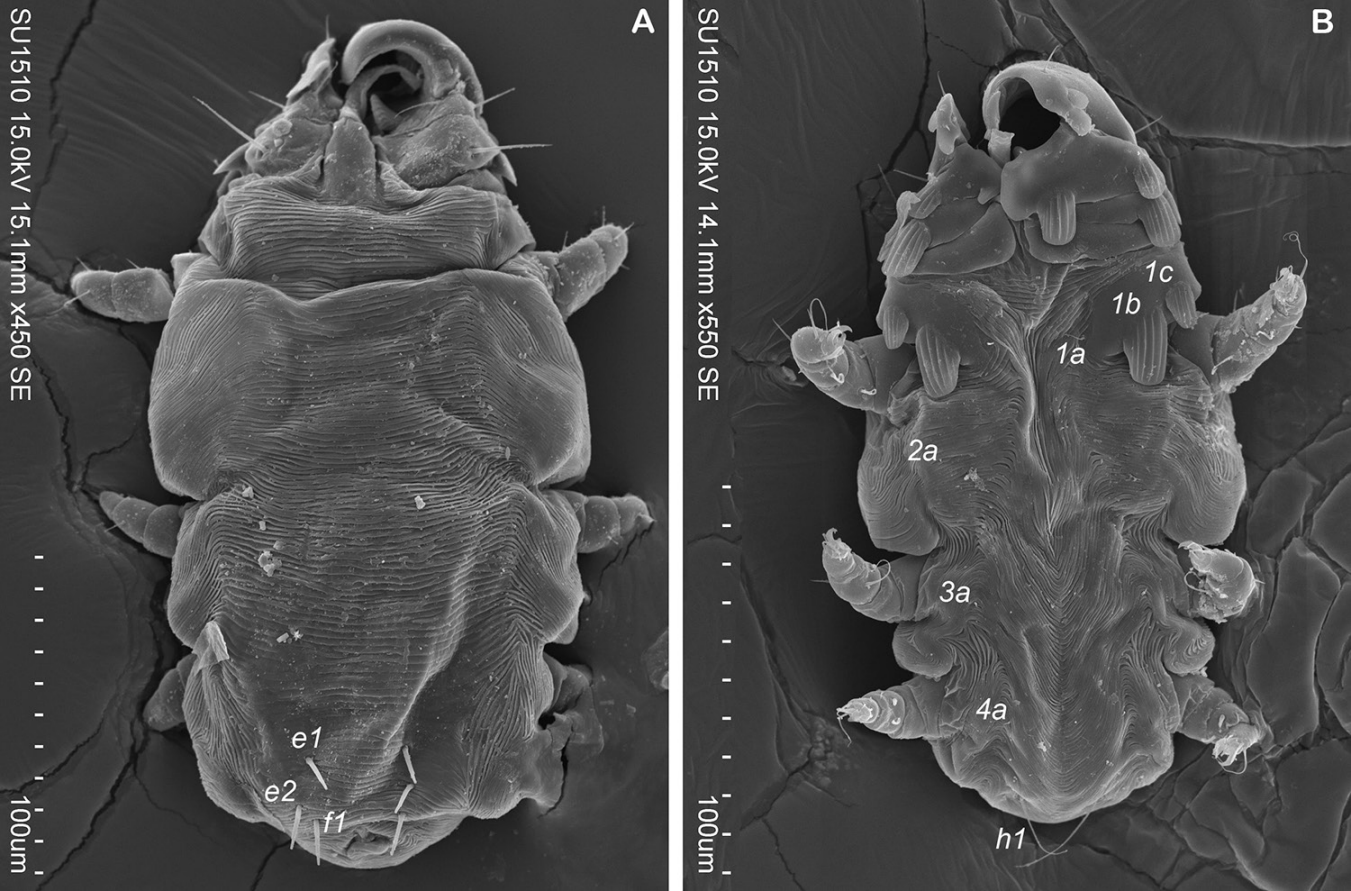
*Ioanella mimon*, tritonymph. **A** Dorsal view **B** Ventral view.

**Trytonymph (Based on 4 tritonymphs).** Dorsal idiosoma. Posterior region of dorsum with 3 pairs of cylindrical and toothed setae: *e1* 14 (11–18), *e2* 15 (14–18), *f1* 14 (12–15) (Fig. 4A). Setae *ve*, *vi*, *sce*, *sci*, *c1*, *d1*, *d2* absent. Ventral idiosoma. Setae *h1* very thin. Setae *2a*, *3a*, *4a* present and minute. Setae *1b* and *1c* shell-shaped, setae *1a* very thin (Fig. 4B). Legs. Tarsi II–IV with 2-1-1 claws. Legs I unequal in shape (Fig. 4B); clasping process with internal striations (Fig. 4B). Setation for legs II–IV: Tarsi 6-6-6, tibiae 5-4-3, genua+femur 2-0-0, trochanters 0-0-0. Number of shell-shaped setae on legs I as follows: 2-0-1-2-1 (Tibia+Tarsus) (Fig. 4B).

###### Remarks.

The male described in this study was determined as part of the genus *Ioanella* by the presence of legs I with the tibia and tarsus fused forming a small complex devoided of apical claws, legs II–IV with two claws, *vi* and *sci* thin and short, all intercoxal setae very short and the lacking of *f2* (Fain 1978). The tritonymph was characterized by the presence of legs I unequal in shape and legs II–IV with 2-1-1 claws (Fain 1978).

The identification of males and tritonymphs as *Ioanella mimon* was done correlating the presence of females on the same analyzed bats considering that myobiids exhibit high specificity to their hosts (Fain 1994).

Comparing our male specimens with the female described by Fain (1973), the only differences observed were in relation to femur and genua III due to we reported three setae instead of two and three setae instead of four, respectively.

This work represents the first description of a male of the genus *Ioanella*, and the second that describes a tritonymph for the genus; previously Fain (1973) described the tritonymph of *Ioanella chrotopterus* (Fain, 1973).

*Eudusbabekia mimon* and *Ioanella mimon* are two species of myobiids recorded originally parasitizing to *Mimon bennettii*, in this work both species are referred for the first time in association with *Mimon cozumelae*, species formerly included as subspecies of *Mimon bennettii* (Ortega and Arita 1997, Villa-Ramírez 1967, Hall 1981), but considered by McCarthy (1987) and Wilson and Reeder (2005), as valid species.

Recent studies suggest that there is no sufficient morphological evidence to maintain *Mimon cozumelae* in a specific level (Gregorin et al. 2008; Hoppe and Ditchfield 2015).

On the other hand, Hurtado and Pacheco (2014) suggested that the genus *Mimon* is not a monophyletic taxon. They proposed to elevate to a genus category the two subgenera (*Mimon* and *Anthorhina*) referred by Gardner and Patton (1972). In accordance with Hurtado and Pacheco (2014), the genus *Mimon* must include to *Mimon bennettii* and *Mimon cozumelae*, and the genus *Gardnerycteris* (=*Anthorhina*) to *Gardnerycteris crenulatum* (É. Geoffroy, 1803) and *Gardnerycteris koepckeae* (Gardner and Patton, 1972). In this context, *Eudusbabekia mimon* and *Ioanella mimon* will be associated with the bat species of the genus *Mimon*, while *Eudusbabekia anthorhinae* Dúsbabek and Lukoschus, 1974 and *Ioanella martae* Dúsbabek and Lukoschus, 1973 to the species of the genus *Gardnerycteris*.

Considering of degree of specificity of myobiid mites to genera or groups of species of hosts (Fain 1994), the referred association could support the Hurtado and Pacheco´s proposal.

###### Distribution.

Guyana (Bartica), Mexico (Yucatan).

## Supplementary Material

XML Treatment for Eudusbabekia mimon

XML Treatment for Ioanella mimon
